# Identification of* ITGA2B* and* ITGB3* Single-Nucleotide Polymorphisms and Their Influences on the Platelet Function

**DOI:** 10.1155/2016/5675084

**Published:** 2016-11-14

**Authors:** Qian Xiang, Shun-Dong Ji, Zhuo Zhang, Xia Zhao, Yi-Min Cui

**Affiliations:** ^1^Department of Pharmacy, Base for Clinical Trial, Peking University First Hospital, Beijing 100034, China; ^2^Jiangsu Institute of Hematology, Key Laboratory of Thrombosis and Hemostasis of Ministry of Health, The First Affiliated Hospital of Soochow University, Suzhou 215006, China; ^3^Collaborative Innovation Center of Hematology, Soochow University, Suzhou 215006, China

## Abstract

The aim of the study was to investigate* ITGA2B* and* ITGB3* genetic polymorphisms and to evaluate the variability in the platelet function in healthy Chinese subjects. The genetic sequence of the entire coding region of the* ITGA2B* and* ITGB3* genes was investigated. Adenosine diphosphate-induced platelet aggregation, glycoprotein IIb/IIIa content, bleeding time, and coagulation indexes were detected. Thirteen variants in the* ITGA2B* locus and 29 variants in the* ITGB3* locus were identified in the Chinese population. The rs1009312 and rs2015049 were associated with the mean platelet volume. The rs70940817 was significantly correlated with the prothrombin time. The rs70940817 and rs112188890 were related with the activated partial thromboplastin time, and* ITGB3* rs4642 was correlated with the thrombin time and fibrinogen. The minor alleles of rs56197296 and rs5919 were associated with decreased ADP-induced platelet aggregation, and rs55827077 was related with decreased GPIIb/IIIa per platelet. The rs1009312, rs2015049, rs3760364, rs567581451, rs7208170, and rs117052258 were related with bleeding time. Further studies are needed to explore the clinical importance of* ITGA2B* and* ITGB3* SNPs in the platelet function.

## 1. Introduction

Platelet aggregation plays a central role in the pathogenesis of acute thrombosis in coronary heart disease, stroke, and peripheral arterial disease. The cellular events leading to platelet aggregation are mediated by the binding of fibrinogen to the glycoprotein (GP)IIb/IIIa receptor of platelets as a final common pathway. GPIIb/IIIa is a platelet-specific, surface membrane receptor and also called alpha IIb beta 3 (aIIb *β*3) in the integrin nomenclature and thus plays a primary role in both platelet adhesion and thrombus formation at the vascular injury site [1]. A large interindividual number variability for GPIIb/IIIa receptors expressed on the platelet surface is commonly observed [2, 3]. Moreover, a defect in the GPIIb/IIIa complex or a qualitative abnormality of this complex is seen in Glanzmann's thrombasthenia patients with impaired platelet aggregation and increased bleeding [4].

The* ITGA2B* gene encodes the aIIb subunit (GPIIb), whereas the* ITGB3* gene encodes *β*3 (GPIIIa). The* ITGA2B* spanning 17 kb has 30 exons, whereas the* ITGB3* spanning 46 kb has 15 exons; they are closely located on chromosome 17q21.32 without evidence for coordinated expression [5]. A few studies have linked single-nucleotide polymorphisms (SNPs) in* ITGA2B* and* ITGB3* with increased or decreased platelet responses to various agonists and the risk of acute coronary syndrome and atherosclerosis [6–8].

A common polymorphism in the* ITGB3*, known as the human platelet antigen 1 (HPA-1b, PlA2, or rs5918), arises from a single-nucleotide change at position 1565 in Exon2 of* ITGB3*, resulting in a leucine (PlA1) to proline (PlA2) substitution at residue 33 [9]. The SNP has been extensively studied as an inherited risk factor for acute coronary syndrome and for its effect on the platelet function [6–8]. The HPA-3 (rs5910) polymorphism results from a thymine to guanine base change that leads to the replacement of isoleucine (HPA-3a) by serine (HPA-3b) at codon 843 of GPIIb (*ITGA2B*). This polymorphism may potentially influence the activity of the GPIIb/IIIa complex and associate with thrombosis [10].

According to the aforementioned studies, GPIIb/IIIa was believed to be a platelet-platelet contact receptor playing an important role in platelet aggregation, and its SNPs were associated with platelet hyperreactivity and have an effect on the pharmacodynamics of antiplatelet drugs. However, limited information was available on other genetic polymorphisms of* ITGA2B* and* ITGB3* in Asian populations or their association with the platelet function [11, 12]. Moreover, the prevalence of the PlA2 allele (rs5918) is dependent on ethnicity, with a frequency of approximately 15/100 in Caucasian populations [13] falling to less than 1/100 in Oriental populations [12, 14].

In this study, regions of the exons of* ITGA2B* and* ITGB3* genes were sequenced in 86 unrelated healthy Chinese. In addition, the association between genetic variants of* ITGA2B* and* ITGB3* exons and the adenosine diphosphate- (ADP-) induced platelet aggregation, GPIIb/IIIa content, bleeding time, and coagulation indexes was investigated in 55 of the 86 subjects.

## 2. Materials and Methods

### 2.1. Study Design

The research was conducted in compliance with the Declaration of Helsinki. The study protocol was approved by the Ethical Review Board of the Peking University First Hospital. All subjects gave their written informed consent prior to participation in the study. Healthy native Chinese subjects (*n* = 86) between 18 and 45 years of age with a body mass index (BMI) of 19–24 kg/m^2^ were included in the study, and their genotypes were unknown.

All the subjects were considered healthy on the basis of their medical history, physical examination, vital signs (blood pressure, pulse rate, and temperature), safety laboratory tests (blood chemistry, hematological tests, and urinalysis), and 12-lead electrocardiography.

The ADP-induced platelet aggregation (transmission, *T*% max), GPIIb/IIIa content (GPIIb/IIIa per platelet), bleeding time, and coagulation indexes, including the mean platelet volume (MPV), platelet count (PLT), prothrombin time (PT), activated partial thromboplastin time (aPTT), fibrinogen (FIB), and thrombin time (TT) were detected in 55 of the 86 subjects. None of the donors had taken any medication for 2 weeks before blood collection.

### 2.2. ADP-Induced Platelet Aggregation

Blood was collected in 3.8% sodium citrate tubes, and platelet-rich plasma (PRP) was obtained by centrifuging blood at 1000 revolutions per minute (rpm) for 10 minutes at room temperature. The PRP was collected in a fresh tube and platelet was counted by Platelet Counter PL100 (Sysmex, Kobe, Japan). Platelet-poor plasma (PPP) was obtained by centrifuging the remaining blood at 3000 rpm for 10 minutes at room temperature, and platelet numbering PRP was adjusted to 250 × 10^9^/L by PPP. Detection was completed within 1 hour after sampling.

Platelet aggregation was determined by the turbidimetric method using 20 umol/L ADP as the agonist. After zero setting with the PPP, assays were performed in platelet-rich plasma in a Chrono-Log aggregometer. Platelet aggregation was quantified as the maximum change in light transmission occurring within 5 minutes of addition of agonist.

### 2.3. GPIIb/IIIa Content

GPIIb/IIIa on the platelet surface was evaluated in the PRP as the maximal binding of a ^125^I-labeled GPIIb/IIIa integrin antiplatelet antibody F(ab)_2_ [15]. The PRP was fixed with equal volume of anticoagulant-fixative solution (10 mmol/L EDTA-Na_2_, 0.2% glutaraldehyde, and 0.02% sodium azide dissolved in PBS, pH 7.4). After stored at 4°C, overnight, platelet was washed by Tyrode's solution twice and resuspended in Tyrode solution, containing 0.35% bovine serum albumin (concentration adjusted to 1 × 10^11^ platelets/L), and sodium azide was added at final concentration of 0.02% (V/V) to preserve the platelet at 4°C for a week-long before assay. For detection, the platelet suspension (100 *μ*L) was added to a 0.5 mL Eppendorf tube and then incubated with 100 *μ*L of antibody (containing 20,000 cpm of labeled monoclonal antibody (McAb) and 0.8 *μ*g of unlabeled McAb) at 37°C for 1 hour, after washed with 0.35% bovine serum albumin/Tyrode's solution for three times. The radioactivity count of the precipitate in the tube was measured. Assuming that one monoclonal antibody would only bind one GP molecule on the platelet membrane, the number of GP molecules on the platelet membrane was calculated as number of GP molecules/platelet = (binding rate × amount of antibody (g) × Avogadro's number)/(molecular weight of antibody × platelet count).

### 2.4. Bleeding Time

The skin bleeding time was carried out using the Simplate-II device (General Diagnostics, NJ, USA). A sphygmomanometer cuff was inflated on the patient's arm to 40 mmHg. The arm was supported at the level of the heart, and a muscular area on the volar aspect distal to the antecubital area was identified and swabbed with alcohol. The Simplate-II device was used to perform the incision perpendicular to the antecubital fossa. Blood was blotted with sterile filter paper every 30 seconds until blood no longer stained the paper. The time from incision to stopping of bleeding was recorded.

### 2.5. Genetic Analysis

Genomic DNA was extracted from peripheral whole blood samples of each subject using a DNA Purification Kit (Wizard; Promega, WI, USA). After the polymerase chain reaction (PCR) products were obtained, all samples were directly sequenced to determine the SNPs in* ITGA2B* and* ITGB3* (Life Technologies Biotechnology Co., Ltd., China).

Primers required for PCR amplification and sequencing were designed according to the wild-type* ITGA2B* and* ITGB3* sequences reported in the GenBank (NG_008331.1 and NG_008332.1, resp.). Tables 1 and 2 describe the primer pairs used to amplify the promoters, the 5′- and 3′-untranslated regions (UTRs), the entire coding regions and the intron-exon junctions of the* ITGA2B* and* ITGB3* genes.

### 2.6. Data Analysis

The Variant Reporter v1.1 (Life Technologies Biotechnology Co., Ltd.) software suite was used for the initial analysis of the sequence, including base calling, fragment assembly, SNP, and sequence insertions/deletions detection. Polymorphisms of* ITGA2B* and* ITGB3* genes were named according to the genomic reference sequences NG_008331.1 and NG_008332.1, respectively. Novel SNPs were named as* ITGA2B*-number or* ITGB3*-number in the present study.

### 2.7. Statistical Analysis

The association between two parameters was assessed by Pearson's two-tailed test. Statistical analyses were performed using the Statistical Package for Social Sciences software program for Windows (SPSS version 16.0). A *P* value of 0.05 was considered significant.

## 3. Results

### 3.1. *ITGA2B* and* ITGB3* Variations

Within the Chinese sample, 13 and 29 variants in* ITGA2B* and* ITGB3* were identified, respectively. Two novel* ITGB3* SNPs with MA(F) > 0.02 were found, which were not reported in the National Center for Biotechnology Information (NCBI) dbSNP database (https://www.ncbi.nlm.nih.gov/snp/). The allele frequencies and Hardy–Weinberg equilibrium test results of the identified polymorphic sites are shown in Tables 3 and 4.

### 3.2. Linkage Disequilibrium Analysis

**Figure 1:**
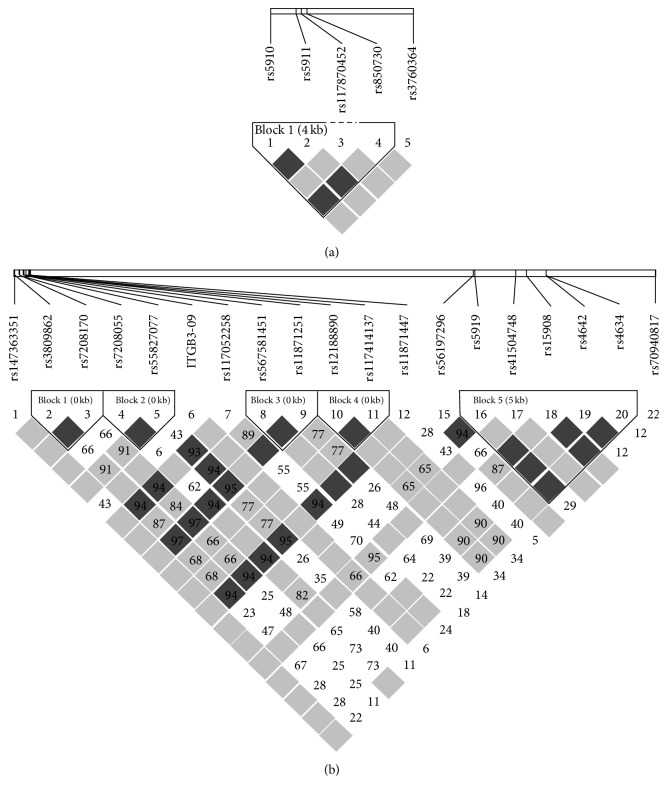
Linkage disequilibrium (LD) map of* ITGB2A* and* ITGB3* SNPs along with their locations in the* ITGB2A* and* ITGB3* genes. Variants present at >1% frequency were included in the LD analysis using the statistics |*D*′| and *r* ^2^. Red depicts a significant linkage between an SNP pair. Numbers inside the square indicate *r* ^2^ × 100. (a)* ITGB2A*. (b)* ITGB3*.

Using the detected polymorphisms greater than 0.01 in frequency [MA(F) > 0.02], linkage disequilibrium (LD) was analyzed for |*D*′| and *r* ^2^ values (Figure 1). For* ITGA2B*, rs5911 was completely linked with rs850730 (*r* ^2^ = 1.00), and rs5910 was strongly linked with rs850730 and rs5911 (*r* ^2^ = 0.95). For* ITGB3*, complete LD was observed between rs4642 and rs4634 (*r* ^2^ = 1.00). Other LD results are shown in Figure 1. A new SNP* ITGB3*-09 was found for which MA(F) in this study was 0.402.

The Hardy–Weinberg equilibrium *P* values of rs1009312 and rs2015049 were <0.001. The possible reason is that the sample in this study may have a Chinese population of other ethnic groups besides the Han population. Those two SNPs were not included in the LD analysis.

### 3.3. Correlation of ADP-Induced Platelet Aggregation, GPIIb/IIIa Content, Bleeding Time, and Coagulation Indexes

**Figure 2:**
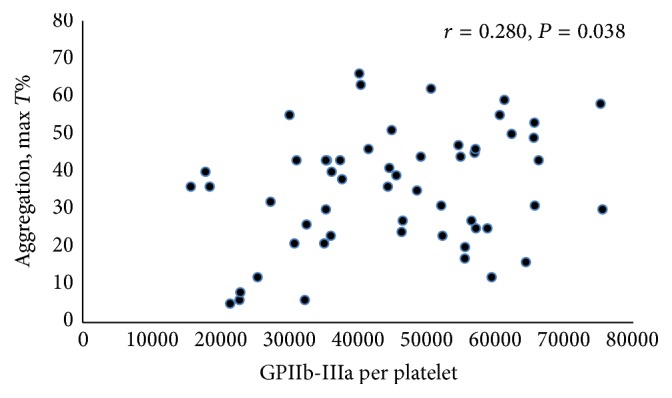
Correlations of the maximal level of ADP-induced platelet aggregation and GPIIb/IIIa content in healthy volunteers. *r*, coefficient of correlation; *P*, significance of correlation (Pearson's test here and elsewhere).

The GPIIb/IIIa contents, present as average numbers of GPIIb/IIIa receptor in each platelet, were associated with ADP-induced platelet aggregation (*r* = 0.280, *P* = 0.038), PLT (*r* = −0.522, *P* < 0.001), PT (*r* = 0.453, *P* = 0.001), and TT (*r* = 0.545, *P* < 0.001) (Figures 1 and 2). The relationship between GPIIb/IIIa content and APTT was not significant (*r* = 0.242, *P* = 0.075), while the association between ADP-induced platelet aggregation and APTT was moderately significant (*r* = 0.267, *P* = 0.049) (Figure S1). The bleeding time had no association with other parameters. It had a negative association trend with MPV but was not significant (*r* = −0.244, *P* = 0.073). The PLT was associated with PT (*r* = −0.415, *P* = 0.002) and TT (*r* = −0.363, *P* = 0.007). The FIB was negatively correlated with TT (*r* = −0.409, *P* = 0.002).

### 3.4. Impact of* ITGA2B* and* ITGB3* SNPs on ADP-Induced Platelet Aggregation, GPIIb/IIIa Content, Bleeding Time, and Coagulation Indexes

For* ITGA2B*, only one SNP rs3760364 was related with the bleeding time (*P* = 0.038) (Table 4). No significant correlation or trend was observed between* ITGA2B* SNPs and other parameters.

For* ITGB3*, the mean values of ADP-induced platelet aggregation of homozygous mutant genotypes in rs56197296 and rs5919 were lower than those of heterozygous mutations and wild type. Moreover, the GPIIb/IIIa per platelet was associated with rs55827077. A trend of GPIIb/IIIa per platelet was observed among rs70940817 GG (45), GA (9), and AA (1) carriers (43807.7 ± 14157.5, 51086.8 ± 19368.0, 61277, resp., *P* = 0.093), although it did not reach a significant level. The bleeding time was significantly related with rs3760364, rs7208170, rs567581451, and rs117052258 (Table 4).

In the present study, SNPs related with PLT were not found.* ITGB3* rs1009312, which is completely linked with rs2015049, was significantly associated with the MPV (Table 4).* ITGB3* rs70940817 was correlated with the PT and APTT (*P* = 0.002 and *P* = 0.003, resp.). The coagulation indexes and their *P* values are summarized in Table 5.

## 4. Discussion

In this study, 13 variants in the* ITGA2B* locus and 29 variants in the* ITGB3* locus in the Chinese population were observed. Two of the 29 variants located in* ITGB3* were novel SNPs with MA(F) > 0.02. Variants in* ITGA2B* and* ITGB3* genes displayed significant interethnic differences in the global populations (Table 6). The C allele frequency of SNP rs5918 (T/C), located in the* ITGB3* gene, was only inhomogeneously distributed at 0.7% in the Chinese population, while it is common in whites and Africans (allele frequency 13.7% versus 12.8%). The following alleles of SNPs, rs7208170A, rs55827077C, rs11871251A, rs1009312G, and rs2015049G, were found to be more common in the Han Chinese and Africans (37%~50%) than in the European ancestry (10%~20%). In the* ITGA2B* gene, the rs5911 was completely linked with rs850730 and strongly linked with rs5910 in the Chinese population in this study, and all the three SNPs were common in whites and Asians. However, the rs5911 was not found in Africans. According to the ethnicity difference described in the preceding text, resequencing of the* ITGA2B* and* ITGB3* genes in the Chinese population was believed to be very important to reveal the function of SNPs.

Variation in the MPV or PLT can have a profound impact on differences in the platelet function between individuals [16, 17], and these traits have a strong genetic component [18–21]. However, limited information is available for Asians. In the present study, the A alleles of rs1009312 and rs2015049, located in* ITGB3*, were positively associated with the MPV (*P* = 0.029). The MPV had a trend in rs7208170 alleles but did not reach a significant level (*P* = 0.051). The difference between PLT and SNPs in* ITGA2B* or* ITGB3* was not significant.

The APTT and PT are clinical tests commonly used to indicate coagulation factor deficiencies [22, 23], activated coagulation, and risk of venous thromboembolism [24, 25]. To date, two genome-wide association studies (GWAS) of APTT and PT conducted in the European ancestry have been reported. One study identified genome-wide significant associations of APTT and variants at F12 (MIM 610619), KNG1 (MIM 612358), and HRG (MIM 142640) [26]. In the other research, the GWAS for APTT and PT was conducted and replicated genome-wide significant associations at KNG1, HRG, F11, F12, and ABO for APTT and identified significant associations at the F7 and PROCR/EDEM2 regions for PT. Eight genetic loci accounted for ~29% of the variance in APTT, and two loci accounted for ~14% of the variance in PT [27]. In this study, the association of APTT, PT, FIB, and TT with* ITGA2B* and* ITGB3*SNPs was investigated. The A allele of* ITGB3* rs70940817 was found to significantly correlate with the elevated APTT and PT (*P* = 0.003 and *P* = 0.002, resp.). The T allele of* ITGB3* rs112188890 was related with APTT (*P* = 0.029), and the G allele of* ITGB3* rs4642 was associated with TT and FIB (*P* = 0.015 and *P* = 0.029, resp.).

The ADP-induced platelet aggregation test was often used to identify the platelet function and the efficacy of antiplatelet drugs. Jones et al. detected 1327 SNPs and investigated their correlation with ADP or collagen-related peptide-induced platelet aggregation in 500 healthy northern European subjects. This identified 17 novel associations with the platelet function (*P* < 0.005) accounting for approximately 46% of the variation in response [28]. In this study, the minor allele of rs56197296 and rs5919 was found to be associated with the decreased ADP-induced platelet aggregation (*P* = 0.014 and *P* = 0.015, resp.).

GPIIb/IIIa is the central receptor of platelet aggregation. The variations of GPIIb/IIIa amount expressed on a platelet surface might affect the platelet-aggregating activity. Many studies reported that the rs5918 correlated with the GPIIb/IIIa receptor expression [29]; however, many conflict studies have also been published [3, 30]. O'Halloran et al. investigated whether three polymorphisms of the GPIIIa promoter (−468G/A, −425A/C, and −400C/A) influenced the RNA expression and receptor density in the platelets of patients with cardiovascular disease [3]. They found a threefold variation between the subjects in the number of GPIIb/IIIa receptors expressed per platelet, although no association between the receptor density and the PlA2 or the three promoter polymorphisms was demonstrated. In the present study, rs55827077, which was located at the promoter region of the GPIIIs gene at position −145, was related with GPIIb/IIIa per platelet.

The bleeding time is a test that evaluates the platelet function* in vivo*. A prolonged bleeding time can result from platelet abnormality, Von Willebrand factor deficiency, or vascular disorders such as Ehlers–Danlos syndrome. A disruption of platelet aggregation results in a prolongation of the bleeding time [31]. In this study, the bleeding time was related with rs3760364 of* ITGA2B* and rs7208170, rs567581451, and rs117052258 of* ITGB3* (*P* < 0.05). No previous study reporting on the effect of SNPs on the bleeding time was found. The mechanism of the relationship needs to be further examined. However, a negative trend between the bleeding time and MPV (*r* = −0.244, *P* = 0.073) was observed. Moreover, rs1009312 and rs2015049 were found to significantly associate with both bleeding time and MPV. Further, the minor alleles of rs7208170 and rs567581451, which were significantly related with the bleeding time, had a trend with the MPV among genotypes, although they did not reach a significant level (*P* = 0.051 and *P* = 0.054, resp.). However, no relationship was observed among SNPs, bleeding time, and MPV in rs3760364 and rs117052258.

In this study, a positive correlation of the level of ADP-induced aggregation and GPIIb/IIIa content was detected in healthy volunteers. This correlation is consistent with a previous report. Yakushkin et al. investigated the relationship between the number of GPIIb/IIIa and the level of ADP-induced aggregation in a group of 35 healthy volunteers and found positive and significant correlations between the level of platelet aggregation induced by different ADP doses (from 1.25 to 20 mmol/L) and the number of GPIIb/IIIa [32].

A strong positive correlation was observed between GPIIb/IIIa per platelet and PLT, PT, and TT (*P* < 0.01). Although the TT was strongly correlated with GPIIb/IIIa per platelet (*r* = 0.545, *P* < 0.001), the relationship of SNPs/TT and SNPs/GP (IIb/IIIa) content is not linked. For example,* ITGB3* rs4642 was significantly related with the TT (*P* = 0.015), while the value of GPIIb/IIIa per platelet had no trend among rs4642 genotypes (*P* = 0.789). The other coagulation indexes were the same. Therefore, the relationship between SNPs and GPIIb/IIIa content cannot be deduced from the correlation of coagulation indexes and SNPs.

Some of the significant correlations between SNPs and platelet function parameters in the present study were not reported previously, which may be due to the reason that SNPs with low MA(F) in the European ancestry were not selected as candidate SNPs in previous studies. For example, the GWAS-genotyping platforms lack the coverage of low frequency/rare variants [33]. Moreover, LD might be another reason, if different genetic patterns (haplotypes) are present in different populations [3].

A possible limitation of the current study is the limited number of subjects. After stratification, the number of subjects in each genotypic group was small, thereby limiting further haplotype association analysis and accession of small effects of an SNP with a small minor allele frequency.

## 5. Conclusion

In summary, as ethnicity difference might limit the interpretation of the function of SNPs, resequencing the* ITGA2B* and* ITGB3* genes and investigating their functions in the Chinese population are very important. In the present study, nine SNPs were found to associate with indexes of platelet and coagulation haemostasis. Newer studies are needed, particularly, to further assess the clinical importance of the above-discussed SNPs in disease susceptibility and antiplatelet drugs pharmacodynamics. Further studies should pay more attention to the roles of* ITGA2B* and* ITGB3* SNPs in ethnic variations.

## Supplementary Material

The supplementary table 1 lists the primer sequences used in this study, and the supplementary figure 1 shows the association of coagulation indexes (Platelet Count, Prothrombin Time, Thrombin Time, Partial Thromboplastin Time) with the maximal level of ADP-induced platelet aggregation or GP IIb-IIIa content in healthy volunteers.

## Figures and Tables

**Table 1:** Summary of GPIIb (*ITGA2B*) variations detected in this study.

Sequence number obtained in this study	dbSNP	Location	NC_000017.11	NM_000419.3	Nucleotide change	NP_000410.2 amino acid change	MA(F)	H-W *P*
ITGA2B-01	rs3760364^*∗*^	5′ upstream end	44390436	-963	ctcccaaggg A/T ctcatttaca		T(0.052)	0.609
ITGA2B-02	New	5′ upstream end	44390152	-679	tagaccaagg T/C ccattcacca		C(0.006)	0.957
ITGA2B-03	New	5′ upstream end	44390081	-608	caagacggag G/A aggagtgagg		A(0.006)	0.957
ITGA2B-04	New	Exon3	44385910	354	tgatgagacc C/T gaaatgtagg	Arg108Stop	T(0.006)	0.957
ITGA2B-05	New	Exon13	44380960	1344	ctcccaggtc C/A tggacagccc	Leu438Met	A(0.006)	0.957
ITGA2B-06	rs201355504	Intron13	44380821	1393+58	cttggcactt C/T cagcgaatgt		A(0.006)	0.957
ITGA2B-07	New	Exon14	44380638	1401	tagacctgat C/G gtgggagctt	Ile467Met	G(0.006)	0.957
ITGA2B-08	rs850730	Intron21	44377095	2188-7C>G	ccctcctcat C/G tcccagatag		G(0.477)	0.289
ITGA2B-09	New	Exon23	44376336	2320	cgtgccggtc C/T gggcagaggc	Arg774Trp	T(0.006)	0.957
ITGA2B-10	rs117870452	Exon23	44376322	2334	gcagctccac C/T tgggcctctg	Gln778=	G(0.012)	0.913
ITGA2B-11	rs5911	Exon26	44375697	2621T>G	ggggctgggg A/C tgggcagccc	Ile874Ser	G(0.477)	0.289
ITGA2B-12	rs5910	Exon30	44372421	3063C>T	acccccaggt C/T ggcttcttca	Val1021=	T(0.465)	0.289
ITGA2B-13	New	3′-UTR	44372213	^*∗*^151C>A	gctacccccc C/A tcctgctgcc		A(0.017)	0.869

dbSNP, single nucleotide polymorphism database; H-W *P*, Hardy–Weinberg equilibrium *P* value; MA(F), minor allele (frequency); GP, glycoprotein.

^*∗*^SNPs which related with bleeding time in this study.

**Table 2:** Primer sequences used in this study.

DNA sequence number	Amplified or sequenced region	Forward primer (5′ to 3′)	Reverse primer (5′ to 3′)	Amplified region NC_000017.11	Length (bp)
ITGA2B-1		TCCTCCTCTTCCGCTTACCG	TACTACCACCGTGCTAGTCC	44389728–44390630	848
ITGA2B-2	Exon1	CCAATATGGCTGGTTGAG	AACTTCCCTTACGGCTCA	44389267–44390059	792
ITGA2B-3	Exon1	CCAGTGCAGCTCACCTTCTA	GATGAGGGAAATGGAACAGA	44388253–44389368	1115
ITGA2B-4	Intron1	TATGAACCACTCCACCCT	TTGGCACTCTTGATTCTG	44388169–44389006	837
ITGA2B-5	Exon2, Exon3	ACCGCTGGTTCTTGTTGC	CCTACGGGCGTCTTCTCA	44385649-44386479	830
ITGA2B-6	Exon4–Exon6	TACAGGGCACAGGGAACAATC	AGAGGCTCTGGGAGGACACG	44384990–44385810	820
ITGA2B-7	Exon7, Exon8	TCCTGGCGGCTATTATTTCT	GCACCGACGACATATTCTGG	44384339–44385186	847
ITGA2B-8	Exon9–Exon11	ATTTGCGCCCTTGTCCTC	AGCCGAATCGCCCATAGA	44383538–44384605	1067
ITGA2B-9	Exon12	CCCTCTGTCTCCCTTTCC	CATCCAGTCTCCCACCAA	44383189–44383838	649
ITGA2B-10	Exon13, Exon14	CCTAGTCTCCTGGGATGTTC	TCACGGGTGTCTTGGTCT	44380390–44381163	773
ITGA2B-11	Exon14–Exon17	TAATCGCCAATTCTGACCC	CACATCCCACCTTCTCCTG	44379854–44380682	828
ITGA2B-12	Exon18	ACCCACTGGACTTGTTCATC	TGTGACTTGGCACTAACCC	44379610–44379957	347
ITGA2B-13	Exon19, Exon20	TGGACGACAGAGCGAGAC	GGCCATACCTCGACATTG	44378354–44378989	635
ITGA2B-14	Exon21	CATGTGACAGTCCCTTGA	AAAGTCACTCACCCAAGGA	44377551–44377933	382
ITGA2B-15	Exon22	CTTGGAGGGTGAAGACTGG	CAACTCCTGACCTCCAGTGA	44376833–44377179	346
ITGA2B-16	Exon23–Exon25	CCAGGTCTAACTTCAGTGTG	GCTCTGGCAGGAAGATCTGT	44375629–44376523	894
ITGA2B-17	Exon26	TCCGACCTGCTCTACATCC	CGGGCTTGCTCACATAGTC	44375277–44375913	636
ITGA2B-18	Exon27, Exon28	ATGACCCTCCCTGCATCTC	CACCTTGACACCTGCCTTT	44374500–44375317	817
ITGA2B-19	Exon29	GCACGCATGGTTCAACGT	CCTCCCGAGTAGCTGAGATT	44374025–44374731	706
ITGA2B-20	Exon30	AAAGGCATCCATTTGTGA	TGTTGGTAAGGCTGGTCTC	44371896–44372566	670
ITGB3-1		CAGGAGGTGGAGGATTGT	GCTGGATTCTTGGGACAC	47252637–47253474	837
ITGB3-2	Exon1	CGGTTCAGAGAAGGCATTCAG	GCTCCAAGTCCGCAACTTGA	47253373–47254015	642
ITGB3-3	Intron1	TTGGCGTAGGAGGTGAGTGA	CCGCAGGAAGCCAAGTTGAA	47253929–47254518	589
ITGB3-4	Intron1	TTGGCGTAGGAGGTGAGTGAG	GAAGTTGCAGTGAGCCGAGAA	47253929–47255063	1134
ITGB3-5	Exon2	ATTGGGAAAGTTGGGAAGG	GAAAGGGCAGCAGTGGTT	47274335–47274773	438
ITGB3-6	Exon3	AGGCTGGTCTTGAACTCTTG	CTCCACCTTGTGCTCTAT	47283116–47283612	496
ITGB3-7	Exon4	GGGCTTTCTGGTTTGCTT	CATTTCCCTCCCATTCTC	47284329–47284978	649
ITGB3-8	Exon5	TGTCTGGGTAACTGTGGT	CATCTGCCTACTTTGCTG	47286124–47286725	601
ITGB3-9	Exon6	TCCAAGGACTGGGACTGA	ATGATGCTGCTGCTATGC	47286919–47287483	564
ITGB3-10	Exon7, Exon8	AGCCCAAGCAAGATAAGT	GGAGAAGGCAGTAAGACC	47289597–47290393	796
ITGB3-11	Exon9	AAACTGGGCTCCAATAAC	TGAGGGACTGAAGGTAAAG	47290561–47291406	845
ITGB3-12	Exon10	CAGGGCAGGGAACAACTT	GGATTGGTCCTTATACTCAAAA	47292050–47292720	670
ITGB3-13	Exon11	GAGCAAGTCCTGCCATAC	TCACAGAGTGTCCTCCATAA	47299101–47299890	789
ITGB3-14	Exon12	CAGAAATGGCATAGGGTT	TCTTGCTGAGTCTGTGGG	47300168–47300824	656
ITGB3-15	Exon13	CTTGAATCTAGGCATCGT	GTATTGAACTCCTGACCC	47302581–47303050	469
ITGB3-16	Exon14	CCTCAAGTAGGTCCCAGTG	AACATGACCACCCAAAGC	47307158–47307721	563
ITGB3-17	Exon15	CTCATCTCCTCCTGTTATTT	TGACATTCTCCCAACCTAC	47309941–47310337	396

**Table 3:** Summary of *ITGB3 *variations detected in this study.

Sequence number obtained in this study	dbSNP	Location	NC_000017.11	NM_000212.2	Nucleotide change	NP_000410.2 amino acid change	MA(F)	H-W *P*
ITGB3-01	rs147363351	5′-UTR	47253042	-820	agcttccaga G/A gttttaagtc		A(0.012)	0.913
ITGB3-02	rs3809862	5′-UTR	47253062	-800	ctggggaaga C/T ccagggactc		T(0.424)	0.822
ITGB3-03	rs7208170^*∗*^	5′-UTR	47253393	-469	aaggcattca G/A cagatgtttg		A(0.419)	0.680
ITGB3-04	New	5′-UTR	47253360	-502	tagtgaataa T/A aaaggactga		A(0.023)	0.913
ITGB3-05	rs7208055	5′-UTR	47253461	-401	gtgaatgtgt C/A ccaagaatcc		A(0.221)	0.273
ITGB3-06	rs55827077^*∗*^	5′-UTR	47253717	-145	tagagaagcc G/C gaggggagga		C(0.448)	0.494
ITGB3-07	rs567581451^*∗*^	5′-UTR	47253771	-91	acccaccgcg -/TCCCC tcccctcccc		insTCCCC(0.058)	0.567
ITGB3-08	rs117052258^*∗*^	5′-UTR	47253855	-7	cgcgggaggc G/C gacgagatgc		C(0.227)	0.771
ITGB3-09	New	Exon1	47253882	21	ggccgcggcc C/G cggccgctct	Pro7=	G(0.407)	0.567
ITGB3-10	rs11871251	Intron1	47254061	79+121	ctgggaatgc G/A cgtgtcctgg		A(0.453)	0.771
ITGB3-11	New	Intron1	47254082	79+152	tggcgcggt C/G ggagccggga		G(0.012)	0.913
ITGB3-12	rs112188890^*∗*^	Intron1	47254101	79+161	gagctgggga C/T cttcctggcc		T(0.116)	0.864
ITGB3-13	rs117414137^*∗*^	Intron1	47254192	79+252	aggctgagcg C/G cttcccggcc		G(0.116)	0.864
ITGB3-14	rs11871447	Intron1	47254252	79+312	ccgcgctcac C/G cggggctgcg		G(0.448)	0.918
ITGB3-15	New	Intron1	47254331	79+391	tggggcttcc G/A ggggttgttc		A(0.006)	0.957
ITGB3-16	rs1009312^*∗*^	Intron1	47254774	79+834	ggcacagccc G/A gggttgctgc		G(0.471)	0.000
ITGB3-17	rs2015049	Intron1	47254865	79+925	ggccgcctct G/A cctcagagga		A(0.529)	0.000
ITGB3-18	rs56197296^*∗*^	Intron5	47287025_47287029	778-45_778-41	catggctgaa TTTGT/- tttgtctcct		delTTTGT(0.169)	0.049
ITGB3-19	rs5919^*∗*^	Exon6	47287174	882	ttgtccagcc T/C aatgacgggc	Pro294=	C(0.262)	0.022
ITGB3-20	rs41504748	Intron7	47290145	1036-40	accaccagct T/C cctttggtaa		C(0.070)	0.334
ITGB3-21	rs15908	Exon9	47290971	1143	cttccagctc A/G/T actttagaac	Val381=	C(0.599)	0.006
ITGB3-22	New	Exon10	47292177	1299	gaggctgtcc C/T caggagaagg	Pro433=	T(0.006)	0.957
ITGB3-23	rs4642^*∗*^	Exon10	47292411	1533A>G	cagcaggacga A/G tgcagccccc	Glu511=	G(0.331)	0.214
ITGB3-24	rs13306487	Exon10	47292422	1544	tgcagccccc A/C/G ggagggtcag	Arg515Gln	A(0.012)	0.913
ITGB3-25	rs4634^*∗*^	Exon10	47292423	1545	gcagcccccg G/A gagggtcagc	Arg515=	A(0.331)	0.214
ITGB3-26	New	Exon10	47292528	1659	gcgagtgtga C/Tgacttctcct	Asp550=	T(0.006)	0.957
ITGB3-27	rs149823724	Exon11	47299519	1902	cagatgcctg C/T acctttaaga	Cys634=	T(0.006)	0.957
ITGB3-28	rs11870252	Intron11	47300459	1914-19	ccttaatcac T/C gtgtcctctc		C(0.035)	0.869
ITGB3-29	rs70940817^*∗*^	Exon12	47300524	1960	cctacatgac G/A aaaatacctg	Glu654Lys	A(0.076)	0.432

dbSNP, single nucleotide polymorphism database; H-W *P*, Hardy–Weinberg equilibrium *P* value; MA(F), minor allele (frequency).

^*∗*^SNPs which related with ADP induced platelets aggregation, GPIIb/IIIa content, bleeding time, or coagulation indexes in this study.

**Table 4:** Association between individual ITGA2B and ITGB3 SNPs and the ADP induced platelets aggregation, GPIIb/IIIa content, bleeding time, PLT, and MPV (mean ± SD).

dbSNP	Genotypes	*N*	Aggregation max *T*%	GPIIb-IIIa per platelet	Bleeding time (s)	PLT (10^9^/L)	MPV (fL)
rs3760364	AA	48	36.6 ± 15.3	45937.1 ± 15214.0	281.3 ± 97.4	277.0 ± 70.87	9.48 ± 0.84
	AT	7	27.4 ± 16.1	41060.9 ± 15560.7	364.3 ± 87.3	257.7 ± 83.7	9.51 ± 0.66
*P*			0.148	0.433	0.038	0.512	0.911
rs7208170	GG	18	38.5 ± 16.2	48835.0 ± 13746.0	326.7 ± 100.8	255.1 ± 62.1	9.14 ± 0.74
	GA	24	35.1 ± 14.3	45714.9 ± 15783.1	288.8 ± 86.6	290.6 ± 82.8	9.63 ± 0.80
	AA	13	31.7 ± 17.1	39709.0 ± 15545.1	249.2 ± 109.1	272.1 ± 60.4	9.68 ± 0.84
*P*			0.229	0.105	0.030	0.428	0.051
rs55827077	GG	18	39.9 ± 16.2	49297.7 ± 14047.7	315.0 ± 86.2	253.2 ± 62.6	9.24 ± 0.65
	GC	22	35.1 ± 13.0	47150.7 ± 13706.9	279.5 ± 102.4	291.9 ± 85.5	9.55 ± 0.85
	CC	14	30.6 ± 18.1	36518.1 ± 16661.9	287.1 ± 113.3	280.1 ± 55.2	9.66 ± 0.93
*P*			0.091	0.021	0.401	0.251	0.141
rs567581451	-/-	49	35.0 ± 14.4	45317.6 ± 14892.6	282.2 ± 99.1	277.3 ± 73.7	9.56 ± 0.80
	-/insTCCCC	6	38.5 ± 24.4	45306.8 ± 19136.0	370.0 ± 64.8	252.3 ± 56.8	8.88 ± 0.62
*P*			0.609	0.999	0.040	0.428	0.054
rs117052258	GG	29	36.7 ± 16.3	44811.1 ± 14492.1	319.7 ± 93.6	277.1 ± 79.2	9.37 ± 0.82
	GC	21	37.3 ± 13.3	48026.0 ± 15188.4	268.6 ± 105.4	267.9 ± 59.9	9.58 ± 0.80
	CC	4	18.3 ± 14.8	31964.5 ± 18104.4	232.5 ± 61.8	309.7 ± 84.4	9.70 ± 1.01
*P*			0.165	0.539	0.028	0.764	0.288
rs1009312^a^	GG	19	39.3 ± 15.9	46470.7 ± 13798.6	326.8 ± 84.2	276.4 ± 95.0	9.13 ± 0.61
	GA	22	34.0 ± 14.6	48883.3 ± 14927.5	287.7 ± 101.3	272.7 ± 58.6	9.64 ± 0.88
	AA	14	32.3 ± 16.6	38145.0 ± 16040.8	250.7 ± 105.0	275.0 ± 59.1	9.71 ± 0.82
*P*			0.189	0.160	0.027	0.944	0.029
rs56197296	TTTGT/TTTGT	42	37.7 ± 15.9	45398.1 ± 14564.7	285.0 ± 94.7	280.5 ± 76.3	9.41 ± 0.81
	TTTGT/del	11	30.9 ± 10.2	45262.9 ± 18722.4	330.0 ± 116.2	249.6 ± 57.1	9.78 ± 0.79
	del/del	2	11.5 ± 7.8	43895.5 ± 16431.0	225.0 ± 63.6	288.0 ± 21.2	9.30 ± 1.13
*P*			0.014	0.916	0.695	0.433	0.416
rs5919	TT	31	38.8 ± 16.1	45342.5 ± 14029.1	299.0 ± 96.6	283.7 ± 78.8	9.34 ± 0.84
	TC	16	34.9 ± 12.5	47696.5 ± 17544.9	315.0 ± 105.6	247.7 ± 58.6	9.64 ± 0.73
	CC	8	23.3 ± 13.8	40455.6 ± 15470.6	217.5 ± 67.6	292.9 ± 60.5	9.73 ± 0.85
*P*			0.015	0.634	0.130	0.728	0.148

Note: Pearson's two-tailed test was used to analyze correlation between genotype and parameter. ^a^rs1009312 was completely linked with rs2015049.

**Table 5:** Association between individual *ITGA2B* and *ITGB3* SNPs and the coagulation indexes (mean ± SD).

	Genotypes	*N*	PT (s)	APTT (s)	TT (s)	FIB (g/L)
rs112188890^a^	CC	42	10.9 ± 1.08	31.2 ± 2.13	15.7 ± 2.22	2.52 ± 0.45
	CT	12	11.2 ± 1.35	32.2 ± 2.73	14.7 ± 1.59	2.72 ± 0.44
	TT	1	12.5	36.7	16.5	2.72
*P*			0.126	0.029	0.299	0.173
rs4642^b^	AA	27	10.9 ± 1.20	30.9 ± 2.25	16.1 ± 2.36	2.43 ± 0.42
	AG	21	11.3 ± 1.08	32.0 ± 2.34	15.2 ± 1.71	2.68 ± 0.44
	GG	7	10.4 ± 1.01	32.3 ± 2.79	14.2 ± 1.39	2.76 ± 0.45
*P*			0.770	0.088	0.015	0.029
rs70940817	GG	45	10.7 ± 1.08	31.1 ± 2.19	15.2 ± 2.01	2.52 ± 0.42
	GA	9	11.8 ± 1.02	32.8 ± 2.26	16.8 ± 2.34	2.75 ± 0.54
	AA	1	12.7	37.2	15.75	3.10
*P*			0.002	0.003	0.078	0.069

MPV, mean platelet volume; PLT, platelet count; PT, prothrombin time; APTT, activated partial thromboplastin time; FIB, fibrinogen; TT, thrombin time.

Note: Pearson's two-tailed test was used to analyze correlation between genotype and parameter.

^a^Strong LD was observed between rs112188890 and rs117414137 (*r* ^2^ = 0.99); ^b^completely LD was observed between rs4642 and rs4634.

**Table 6:** Comparison of *ITGA2B* and *ITGB3* allele frequencies in different ethnic groups.

dbSNP		MA(F)^a^			
Number		This study Chinese	Asian^b^	European-ancestry^c^	African^d^
ITGA2B-01	rs3760364	T(0.052)	T(0.012)	T(0)	T(0)
ITGA2B-08	rs850730	G(0.477)	G(0.467)	G(0.297)	G(0.437)
ITGA2B-11	rs5911	G(0.477)	G(0.467)	G(0.305)	G(0)
ITGA2B-12	rs5910	T(0.465)	T(0.442)	T(0.376)	T(0.432)
ITGB3-03	rs7208170	A(0.419)	A(0.496)	A(0.194)	A(0.431)
ITGB3-05	rs7208055	A(0.221)	A(0.225)	A(0.117)	A(0.356)
ITGB3-06	rs55827077	C(0.448)	C(0.417)	C(0.158)	C(0.39)
ITGB3-08	rs117052258	C(0.227)	C(0.208)	NA	NA
ITGB3-10	rs11871251	A(0.453)	A(0.482)	A(0.199)	A(0.373)
ITGB3-12	rs12188890	T(0.116)	T(0)	T(0.023)	T(0)
ITGB3-16	rs1009312	G(0.417)	A(0.434)	A(0.125)	A(0.432)
ITGB3-17	rs2015049	G(0.471)	A(0.478)	A(0.138)	A(0.413)
NF.	rs5918	C(0)	C(0.007)	C(0.137)	C(0.128)
ITGB3-18	rs56197296	delTTTGT (0.169)	NA	NA	NA
ITGB3-19	rs5919	C(0.262)	C(0.232)	C(0.049)	C(0.187)
ITGB3-23	rs4642	G(0.331)	G(0.31)	G(0.283)	G(0.306)
ITGB3-25	rs4634	A(0.331)	A(0.350)	A(0.283)	A(0.331)
ITGB3-29	rs70940817	A(0.076)	NA	NA	NA

dbSNP, single nucleotide polymorphism database; MA(F), minor allele (frequency); SNP, single nucleotide polymorphisms; NF, not found; NA, not available.

^a^Resource of MP(F)s was from GenBank and HapMap, https://www.ncbi.nlm.nih.gov/snp/; ^b^Han Chinese in Beijing, China (CHB), or CHB + Japanese in Tokyo, Japan (JPT), if there is no CHB data in the SNP; ^c^Utah residents with Northern and Western European ancestry from the CEPH collection (CEU); ^d^Yoruba in Ibadan, Nigeria (YRI).
