# Supplementary material for: Identification of ITGA2B and ITGB3 Single-Nucleotide Polymorphisms and Their Influences on the Platelet Function
Source: Biomed Res Int. 2016 Nov 14;2016:5675084. doi: 10.1155/2016/5675084 (PMC5124636; doi:10.1155/2016/5675084)
Supplement: Supplementary file 1 — The supplementary table 1 lists the primer sequences used in this study, and the supplementary figure 1 shows the association of coagulation indexes (Platelet Count, Prothrombin Time, Thrombin Time, Partial Thromboplastin Time) with the maximal level of ADP-induced platelet aggregation or GP IIb-IIIa content in healthy volunteers. [file 5675084.f1.pptx]

## Slide 1
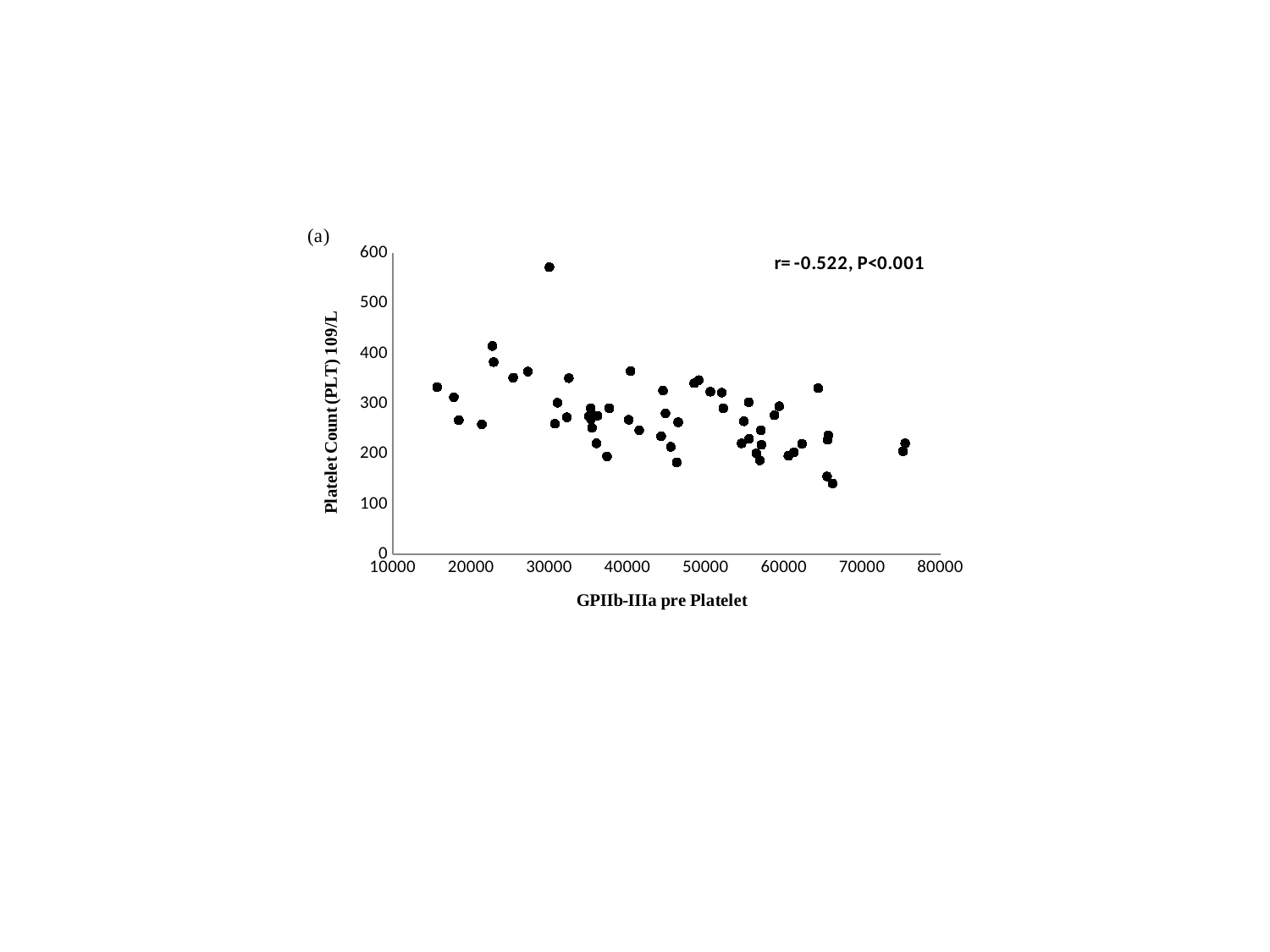

### Chart:
| Category | r= -0.522, P<0.001 |
|---|---|

## Slide 2
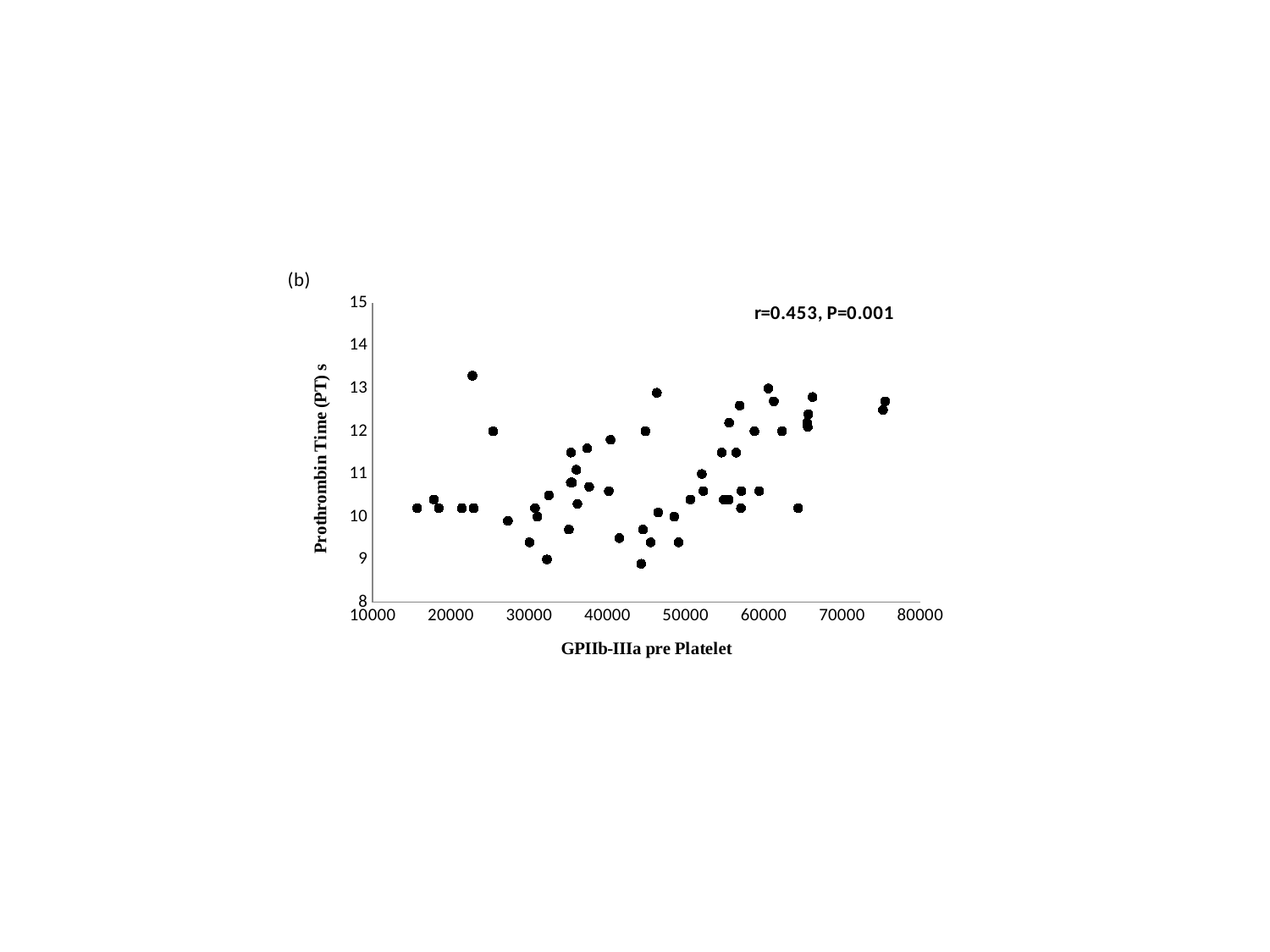

### Chart:
| Category | r=0.453, P=0.001 |
|---|---|

## Slide 3
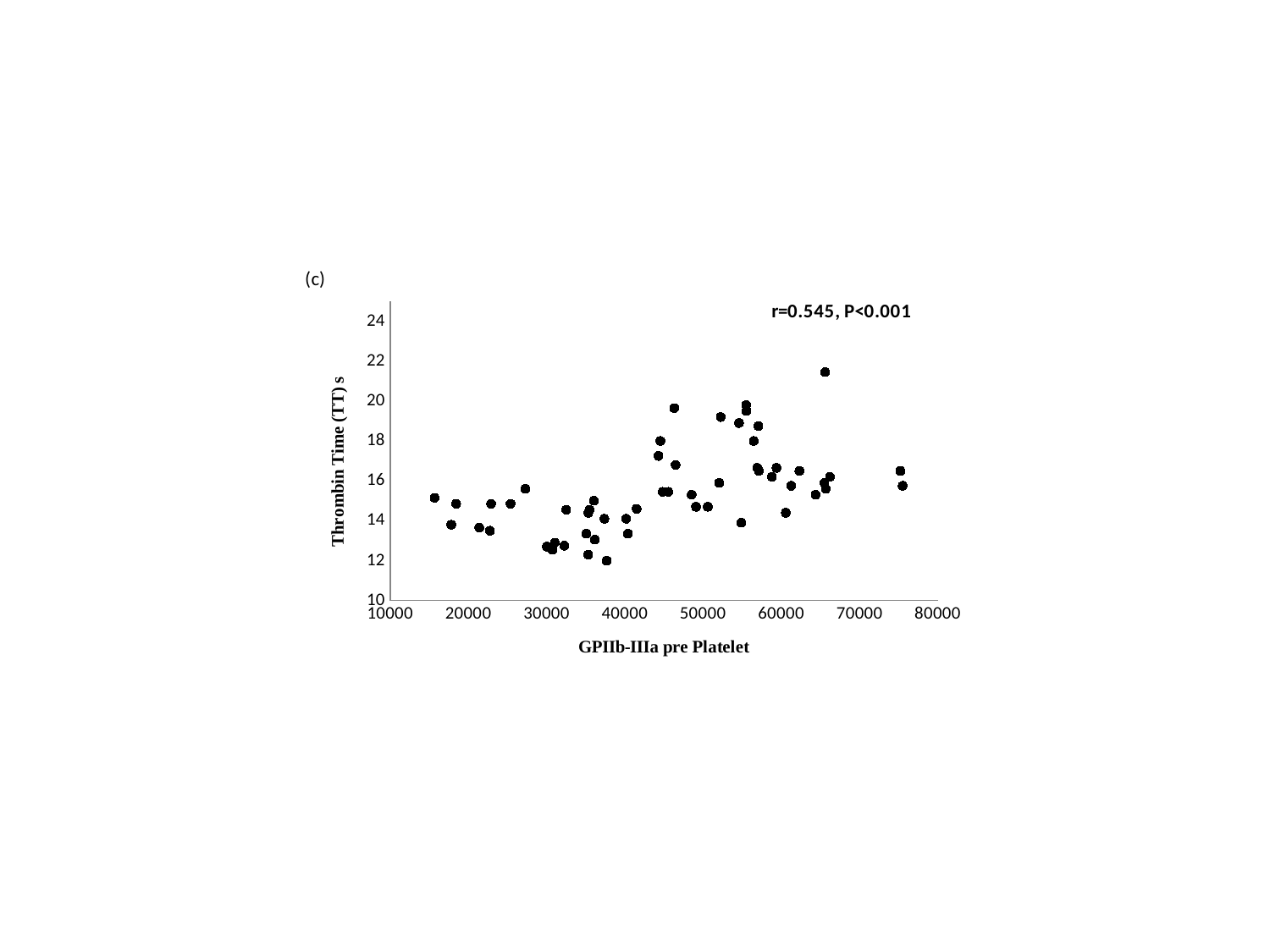

### Chart:
| Category | r=0.545, P<0.001 |
|---|---|

## Slide 4
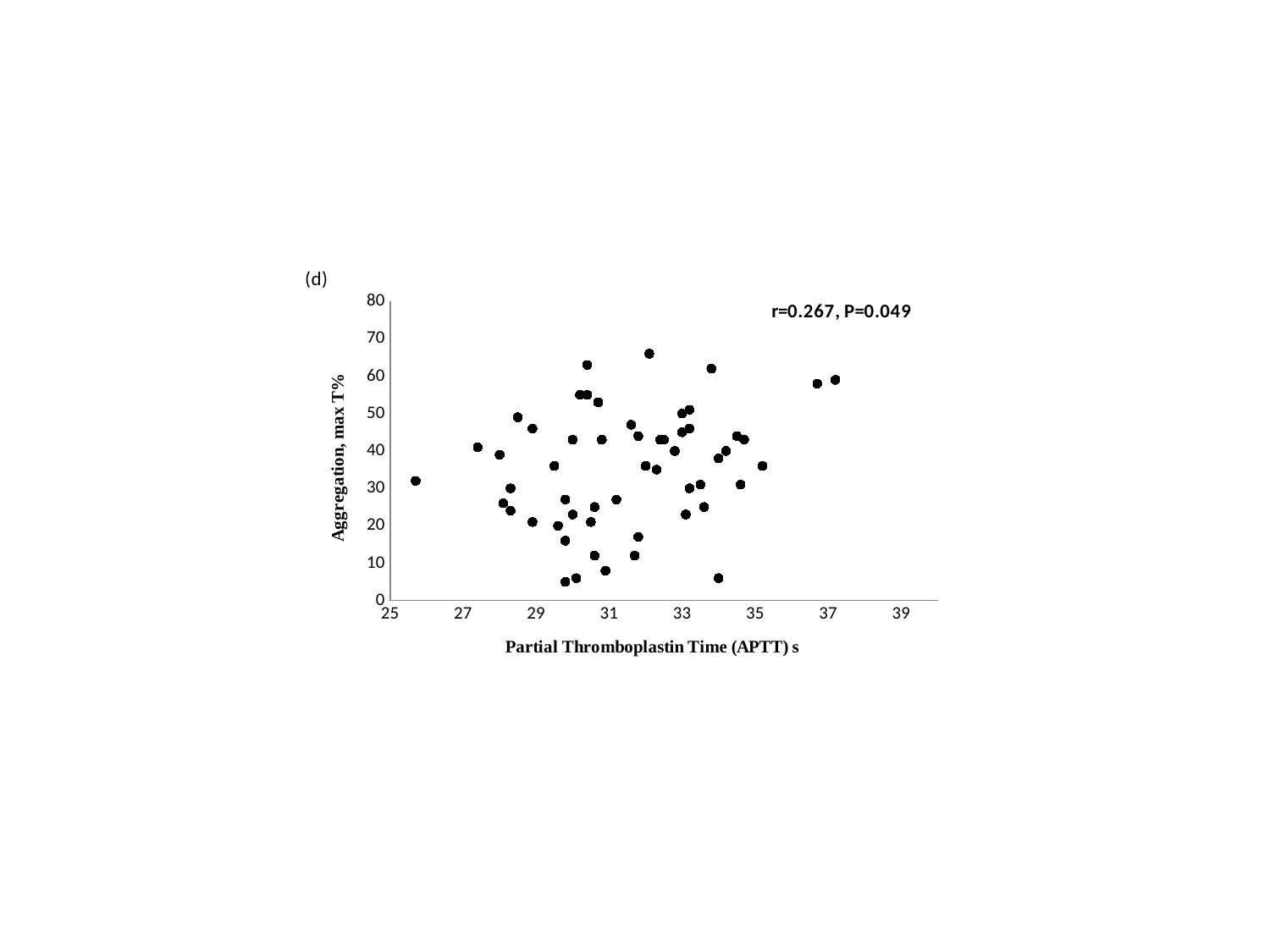

### Chart:
| Category | r=0.267, P=0.049 |
|---|---|
